# Intra-manufacture Diameter Variability of Rotary Files and Their Corresponding Gutta-Percha Cones Using Laser Scan Micrometre

**DOI:** 10.22037/iej.v13i2.14710

**Published:** 2018

**Authors:** Hesam Mirmohammadi, Monika Sitarz, Hagay Shemesh

**Affiliations:** a *Department of Cariology Endodontology Pedodontology, Academic Centre for Dentistry Amsterdam (ACTA), Universiteit van Amsterdam and Vrije Universiteit, the Netherlands; *; b *Department of Endodontics, Shahid Beheshti University of Medical Sciences, Tehran, Iran; *; c *Department of Conservative Dentistry and Endodontics, Medical University of Lublin, Lublin, Poland*

**Keywords:** Diameter, Gutta-Percha, Root Canal, Rotary File, Size

## Abstract

**Introduction:**

Manufacturers offer gutta-percha (GP) cones matched with different sizes of endodontic files as an attempt to simplify the obturation process and create a tight seal in the canal. The purpose of this study was to evaluate whether intra-manufacture GP diameters matched the diameters of their corresponding files at different levels using laser micrometre.

**Methods and Materials:**

Twenty files and corresponding GP master cones of Reciproc R40 (40/0.06) (VDW, Munich, Germany), WaveOne Large(40/0.08)(Dentsply Maillefer, Ballaigues, Switzerland), ProTaper F3 (30/0.09) (Dentsply Maillefer, Ballaigues, Switzerland), and Mtwo 40/0.06 (VDW, Munich, Germany) were examined using laser micrometre (LSM 6000 by Mitutoyo, Japan) with accuracy of 1 nm to establish their actual diameter at D_0_, D_1_, D_3_ and D_6_. The data was analysed using the independent *t*-test. The differences were considered at 0.05.

**Results:**

The diameter of GP master cones was significantly larger than that of the corresponding files at all levels in all brands. ProTaper GP diameter was closest to the file diameter at D1 (GP=0.35, File=0.35 mm), and D3 (GP=0.48, File=0.49).

**Conclusion:**

Within the same manufacturer, GP cone diameters do not match the diameters of their corresponding files. Clinicians are advised to use a GP gauge to cut the tip so as to appropriate the diameter from a smaller sized GP cone.

## Introduction

Successful endodontic treatment is based on correct diagnosis, canal debridement and disinfection, obturation and coronal restoration [1]. Good sealing reduces coronal leakage and bacterial contamination, stops influx of periapical tissue fluids and entombs the remaining irritants and surviving bacteria in the canal [2-6]. The most common method to fill the prepared canal space is the obturation of the root canal system (RCS) with gutta-percha (GP) and sealer [7, 8].

Nickel-titanium rotary files were introduced to endodontics more than two decades ago [9]. These files usually have greater tapers than hand instruments [10]. Nowadays, manufactures offer a plethora of nickel-titanium rotary systems classified by different features (*e.g.* tip-design, cross-section, cutting edge, tapers, diameter, composition, movement). The preferred filling method is the subject of much debate and research. The single cone technique attracted the attention of many investigators owing to its speed and effectiveness [11].

Although a perfect match between instrumented canal and GP cone is impossible, if the size and taper of the master cone differs significantly from the prepared area by the master file, insufficient obturation may result. Ideally, GP cones should closely match the diameter and taper of the last instrument used to the working length [12]. 

Previous studies reported variability in actual sizes of GP [13] and files [10, 14] amongst different endodontic systems. Chesler *et al*. [15] evaluated the diameter and taper of rotary instruments and their corresponding GP cones within the same manufacturer using scanning electron microscopy (SEM). They observed significant differences between the file and the corresponding GP cone regarding to tapers and diameters. However, the use of SEM to evaluate the dimensional change, especially for a thermoplastic material like GP, had its drawbacks. These problems are due to the special thermal and pressure conditions in the SEM chamber that could influence the dimension of GP cones.

A laser-scan micrometer (LSM) system was previously used to measure the diameter changes of different dental materials [16-19]. The device displayed the specimen dimensional data rapidly and accurately. It used a highly directional parallel-scanning laser beam. LSM was a non-destructive, non-contact measuring system, which combined high rate scanning with a highly accurate measurement (0.00001 mm) [20]. A laser beam was directed at a polygonal mirror rotating at high speed in exact synchronism with highly stable pulses from the system clock. The reflected beam was rotating clockwise as it swept across the input surface of a collimating lens. However, as the beam moved or scanned downwards, it changed its direction to be always horizontal after the lens’ exit surface. This horizontal beam entered the measuring space and, with no work piece present, and *via* a condensing lens reached a receiver to produce an output signal. When a simple work piece (a GP cone, for example) was put into the measuring space, the beam would be interrupted for a time during its sweep. This time, as indicated by clock pulses when the receiver signal was absent, was proportional to the work piece dimension in the downward direction [21].

To date, there have been no published papers comparing diameters of nickel-titanium rotary files with their matching GP cones using LSM as a non-destructive method. The aim of this study was to evaluate the diameter of four brands of rotary files and their adjusting GP cones by means of LSM.

## Materials and Methods

The following rotary files and their corresponding GP cones were investigated: Reciproc R40 (40/0.06) (VDW, Munich, Germany) WaveOne Large (40/0.08) (Dentsply Maillefer, Ballaigues, Switzerland), ProTaper F3 (30/0.09) (Dentsply Maillefer, Ballaigues, Switzerland), and Mtwo (40/0.06) (VDW, Munich, Germany). Based on pilot data and a power analysis, it was determined that 20 specimens from each brand would meet the constraints of *α*=0.05 and power=0.80. After receiving the materials, they were conditioned at 23±2^°^C at 50±5% humidity.

**Figure 1:**
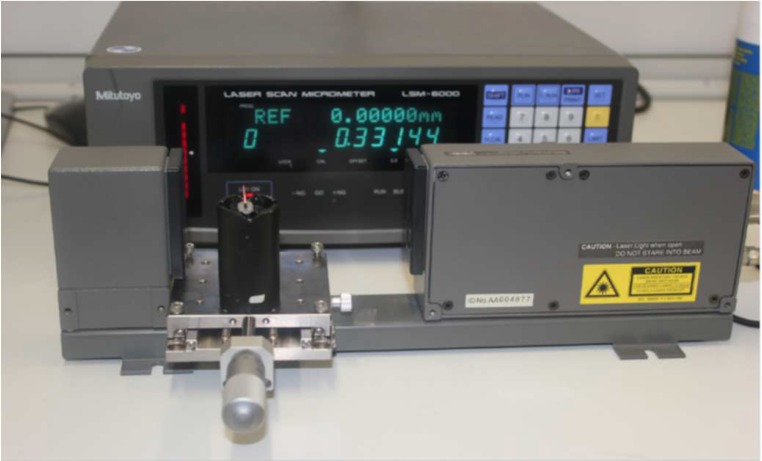
Travel crossed roller table connected to a micrometre perpendicular to the scanning laser beam of a laser scan micrometer. D_0_ level was established as a first reading achieved by LSM, where the specimens touched the laser beam; consequently, specimens were moved manually using micrometre ruler for further measurements

Specimens were randomly assigned a number, from 1 to 160, in order to keep the operator blind during the measurement process. Specimens were mounted on a special jig using prepared impressions of composite (Z250, 3MSPE, Germany). Jig was settled on a travel crossed roller table connected to a micrometre (Mitutoyo, Japan) with an accuracy of 0.1 µm perpendicular to the scanning laser beam of a laser scan micrometre (LSM 6000, Mitutoyo, Japan) in order to obtain accurate reproducible results (Figure 1). Diameters (D) were measured at four levels, 0 mm, 1 mm, 3 mm and 6 mm from the tip of the files or cones. D_0_ level was established as a first reading achieved by LSM, where the specimens touched the laser beam. Consequently, specimens were moved manually using micrometre ruler for further measurements (Figure 1). All the measurements were performed at room temperature 23±2 ^°^C and normal humidity (50±5%). 

***Statistical analysis***

Shapiro-Wilk normality test revealed that data was normally distributed. The comparisons between files and GP cones diameters were analysed with the independent *t*-test using SPSS/PC version 17 (SPSS Inc., Chicago, IL. US). The differences were considered as significant for *P*<0.05 and highly significant for *P*<0.01. 

## Results

The diameters of GP cones were significantly larger than the diameters of corresponding files at all levels for all brands (Table 1). For each tested brand, the independent *t*-test revealed highly significant differences (*P*<0.0001) between GP cone diameter and corresponding file at all measurement levels. The intra-manufacture mean differences for diameter at D_1_ were 0.17±0.04, 0.003±0.01, 0.1±0.003, 0.17±0.005 for Mtwo, ProTaper, WaveOne, and Reciproc respectively. WaveOne

**Table 1:** Diameter measurements of 4 endodontic rotary systems at 4 different levels: 0, 1, 3, and 6 mm

**Diameter**	**0 mm**		**1 mm**		**3 mm**		**6 mm**	
**File/GP size**	File (SD)	GP (SD)^*^	File (SD)	GP (SD)^*^	File (SD)	GP (SD)^*^	File (SD)	GP (SD)^*^
**Mtwo **	0.094 (0.03)	0.286 (0.013)	0.34 (0.073)	0.502 (0.031)	0.47 (0.096)	0.605 (0.03)	0.624 (0,117)	0.79 (0.029)
**ProTaper ** ^X^	0.125 (0.016)	0.187 (0.011)	0.349 (0.008)	0.353 (0.022)	0.477 (0.014)	0.488 (0.03)	0.615 (0.018)	0.67 (0.038)
**WaveOne ** ^X^	0.093 (0.004)	0.256 (0.009)	0.4 (0.024)	0.498 (0.021)	0.539 (0.02)	0.608 (0.031)	0.665 (0,017)	0.76 (0.037)
**Reciproc **	0.095 (0.003)	0.239 (0.012)	0.304 (0.026)	0.475 (0.021)	0.475 (0.028)	0.563 (0.022)	0.633 (0.062)	0.687 (0.023)

X *: Variable taper with no manufacture data;*

* *: The intra-manufacture diameters of GP cones were significantly larger than the corresponding files (P<0.0001)*

Large revealed the best match with the manufacture claimed size at D_1_ (40±0.02). Despite a significant difference between GP cones’ diameters and their corresponding files, ProTaper F3 showed a better match considering the measurements at different levels: D_1_, D_3_ and D_6_.

## Discussion

Previous studies of dimensional variability of GP cones and files used either a measuring microscope, according to the protocol outlined in ANSI/ADA Specifications No. 78, [13, 14] or an SEM according to ANSI/ADA Specifications No. 101. This is the first investigation using LSM to study the diameter variability of rotary systems.

LSM could be used under controlled environmental conditions such as temperature, humidity and pressure. GP cones are partially crystalline viscoelastic polymeric materials and thus, an environmental change may cause a dimensional variation. Hence, the use of SEM could bring flaws into the accuracy of the data, as cones should be saturated and later placed in SEM chamber under a high pressure [22].

**Figure 2:**
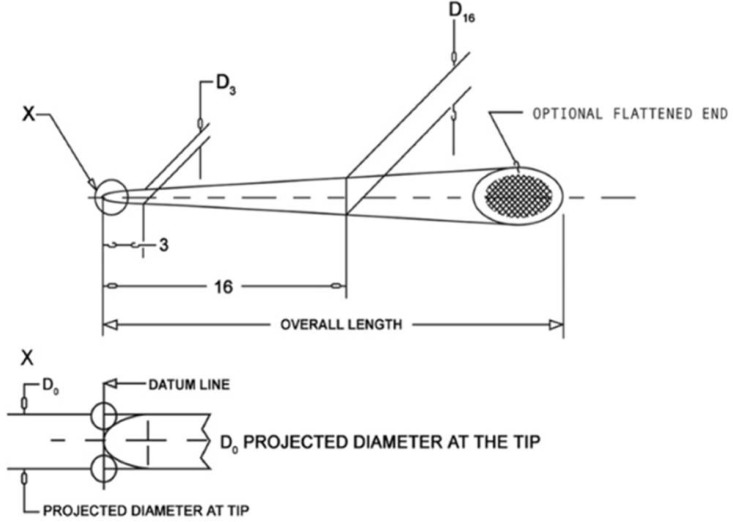
Diagram representation of the tapered sized cones and measurement sites for diameter (D0) (Adapted from ANSI/ADA specification No. 78)

In the current study, the diameter could be measured at 0 mm level using LSM. In a recent article, Chesler *et al*. [15] was unable to measure the diameter at the tip of the files or GP cones under SEM and therefore they provided the data from the D_1_. Two studies reported data for D_0_ using measuring microscope, which met the ANSI/ADA specifications (Figure 2), although it was not the diameter at the tip [9, 10]. Considering the data from the current study, the manufactures’ provided size would actually corresponded to D_1 _and not D_0_. 

It may not be of clinical importance but could need rethinking on definition of D_0_, especially for the manufactures’ reported specifications.

Previous studies investigated the taper of the files, and found that the most examined files had taper measurement smaller than the nominal taper. However, in the current study, tapering of the specimens was not reported, as ProTaper and WaveOne had a variable taper with no clear industry standard.

In this study, at each level, GP cones were always larger than the corresponding file in diameter, which is in agreement with the study by Chesler *et al. *[15]. Although such findings might not be a significant problem for a skilled endodontist, an inexperienced clinician may find it frustrating and time consuming. This is especially true as larger fitting master cones - in comparison with the master file - would result in premature binding or poor adaptability of GP to the canal walls, and consequently shorter fillings. Since the length of the root canal filling is an outcome predictor for endodontic treatments [1, 23], the importance of a well-fitted master cone is obvious. 

The diameter variability of GP cones may be caused by the high plasticity of GP [21-25]. Despite standard procedures throughout manufacturing and packing, mechanical deformation can also occur. Likewise, during transportation and storage due to temperature extremes, shrinkage and/or expansion can result. GP master cones are better kept refrigerated; however, there seems to be a lack of information on the influence of environmental changes such as temperature on GP cones [25].

Variability between nickel-titanium rotary files and GP cone sizes exists within tested manufacturers’ systems. Clinicians should respect individual root canal anatomy and choose a master cone based on the clinical result of the instrumentation, not on the advertised size. Practitioners are advised to check the Master GP cone fit using radiographs. In case of a mismatch, they can use a smaller size tip diameter and a GP gauge to cut the tip to the needed diameter.

## Conclusion

Within the same manufacturer, GP cone diameters do not match the diameters of their corresponding files. Clinicians are advised to use a GP gauge to cut the tip in order to appropriate the diameter from a smaller-sized GP cone.
